# Deformed wing virus affects foraging success and foraging specialization of honeybee workers

**DOI:** 10.1038/s41598-025-31753-0

**Published:** 2025-12-10

**Authors:** Helena Mendes Ferreira, Kristof Benaets, Lina De Smet, Dirk C. de Graaf, Tom Wenseleers

**Affiliations:** 1 Laboratory of Socioecology and Social Evolution, Department of Biology, KU Leuven, Leuven, Belgium; 2 Laboratory of Molecular Entomology and Bee Pathology (L-MEB), Department of Biochemistry and Microbiology, Ghent University, Gent, Belgium

**Keywords:** Deformed wing virus (DWV), Honeybees, Foraging behaviour, Pollinator decline, Bee health, Ecology, Ecology, Zoology

## Abstract

Deformed wing virus (DWV) is a major driver of honeybee colony losses, yet its sublethal effects on adult foraging behaviour remain underexplored. Building on evidence that covert DWV infections impair sucrose responsiveness and associative learning, we tested whether infection changes foraging success and specialization in worker bees. Using a controlled experiment, we marked 1,000 newly emerged workers that were either inoculated with DWV lysate or injected with an RNA-interference control that suppressed viral replication. Foraging activity, success, and specialization were recorded. Our results show that DWV-infected bees began foraging earlier (“precocious foraging”) and had higher mortality, reducing their lifespan as foragers. Furthermore, infected nectar foragers were significantly less likely to return with nectar, and when they did, it contained markedly lower sugar concentrations compared to control bees. Conversely, infected bees were slightly more likely to return with pollen and showed greater specialization in pollen foraging, although pollen load weights were similar between treatments. These findings indicate that DWV disrupts multiple aspects of foraging ecology by accelerating behavioural maturation, shortening forager lifespan, reducing nectar yield and quality, and shifting resource preference towards pollen. Such changes may reflect a compensatory response to impaired nectar collection, but nonetheless could compromise colony nutrition, particularly when high-quality nectar is scarce. Our results align with previous work linking DWV to impaired foraging efficiency, and altered foraging specialization, and reduced honeybee survival, underscoring DWV’s substantial sublethal costs. By reducing nectar foraging while leaving pollen loads unaffected, DWV may limit honey stores and brood rearing during high demand periods, contributing to seasonal losses. Even moderate declines in nectar-foraging can undermine colony resilience, highlighting the need to address DWV’s role in the pollinator crisis.

**Supplementary Information:**

The online version contains supplementary material available at 10.1038/s41598-025-31753-0.

## Introduction

 Honeybees (*Apis mellifera*) are key generalist pollinators of wild plants and crops, and they pollinate roughly one‑third of global food and 90% of animal pollinated crops^1,2^. To meet the increasing demand for agricultural pollination, the number of managed honeybee colonies should at a minimum be maintained or, if possible, increased^3^. In this context, it is alarming that over the past decades, colony losses have significantly increased in Europe^4–6^, North America and Canada^7,8^ which demands for strategies in order to improve honeybee health. The underlying causes of these colony losses are diverse, and current research supports a multifactorial explanation involving interacting stressors, including various pathogens and parasites, malnutrition due to poor floral diversity and the harmful effects of agrochemicals, that often act together, sometimes synergistically^4,5,9–11^. Among these stressors, deformed wing virus (DWV), a positive-stranded RNA virus^12–16^, has been consistently identified as a major contributor to honeybee colony losses and winter mortality in both Europe^17–22^ and the United States^23,24^ and Canada^25^.

DWV continues to pose a significant threat to global honeybee populations^26,27^. DWV is spread by the ectoparasitic mite *Varroa destructor*, a notorious pest that feeds primarily on the fat body tissue of adult bees, pupae and larvae rather than hemolymph as previously thought^28^. DWV originated in Asia, where it initially infected the Eastern honeybee *Apis cerana* and later expanded to infect the Western honeybee *Apis mellifera*, becoming the most widespread honeybee virus globally^29–31^. This virus is now ubiquitous in regions where Varroa mite is present, as this ectoparasite serve as an effective vector for virus transmission^13,26,31–33^.

Deformed wing virus infections of honeybee larvae and pupae, caused by the vectorial transmission via the *Varroa* mite, lead to the characteristic wing deformities after which DWV is named. Additional symptoms of these so-called overt infections are shortened and bloated abdomens, miscolouring and shortened life spans^34–36^. A crucial factor in the development of this pathology is the replication of the virus inside the mite prior to transmission, which leads to highly elevated viral titers upon infection^37^. However, recent findings have shown that this virus can also be transmitted in a non-propagative manner, meaning replication within the mite is not necessary for transmission^33^. In addition to mite-mediated transmission, the virus can spread horizontally via faecal matter, oral contact or cannibalism and vertically through infected sperm or eggs, allowing transmission from parents to offspring^38–43^. Infections resulting from horizontal transmission routes, mites carrying low DWV loads, or occurring in adult honeybees often proceed without visible morphological symptoms^34,36,44^. Despite the absence of visible symptoms, these covert infections can still lead to long-term colony level consequences, including weakness, population decline, and eventual collapse^45^.

DWV infection in adult workers have also been shown to selectively infect the bees’ mushroom bodies^46^, and to lead to specific impairments in sucrose responsiveness and associative olfactory learning^47^, similarly to other common honeybee viruses such as the Israeli acute paralysis virus (IAPV), which also targets neural tissues and alters behaviour^48^. These findings suggest that viruses such as DWV could have a significant sublethal impact on honeybee foraging behaviour and performance, and that even seemingly covert infections in adult workers may nonetheless carry substantial long-term costs under natural conditions, as covert DWV infections have been shown to negatively affect both foraging efficiency and survival^49^.

To better characterize the impact of DWV on honeybee foraging behaviour, we compare the foraging activity, foraging success and foraging specialization of marked DWV-infected and uninfected control bees. These two treatment groups were established using a previously developed method in which one group of adult bees was artificially inoculated with DWV lysate while the other group was injected with an RNAi treatment that partially protected the bees against the virus^49,50^. Daily observation and sampling of returning foragers of both groups subsequently allowed us to determine the type and yield of the collected forage by weighing pollen and measuring the sugar content of the nectar they collected, thereby providing us with a good measure of the nectar quality. Our results confirm the theory that DWV infections strongly affect honeybee foraging behaviour, inducing mortality and shortening the lifespan of honeybee foragers, and interfere with the workers’ ability to engage in nectar foraging and collect high quality nectar, likely due to the viruses’ effect on the bees’ sucrose responsiveness. These results show that DWV infections indeed carry significant costs and impair the honeybees’ normal abilities to efficiently engage in foraging. These results may explain the observed link between DWV infection and colony losses and winter mortality, documented in several previous studies^12,14,16,21,24,25,36,51^.

## Results

### Effects of deformed wing virus on observed foraging activity

**Fig. 1:**
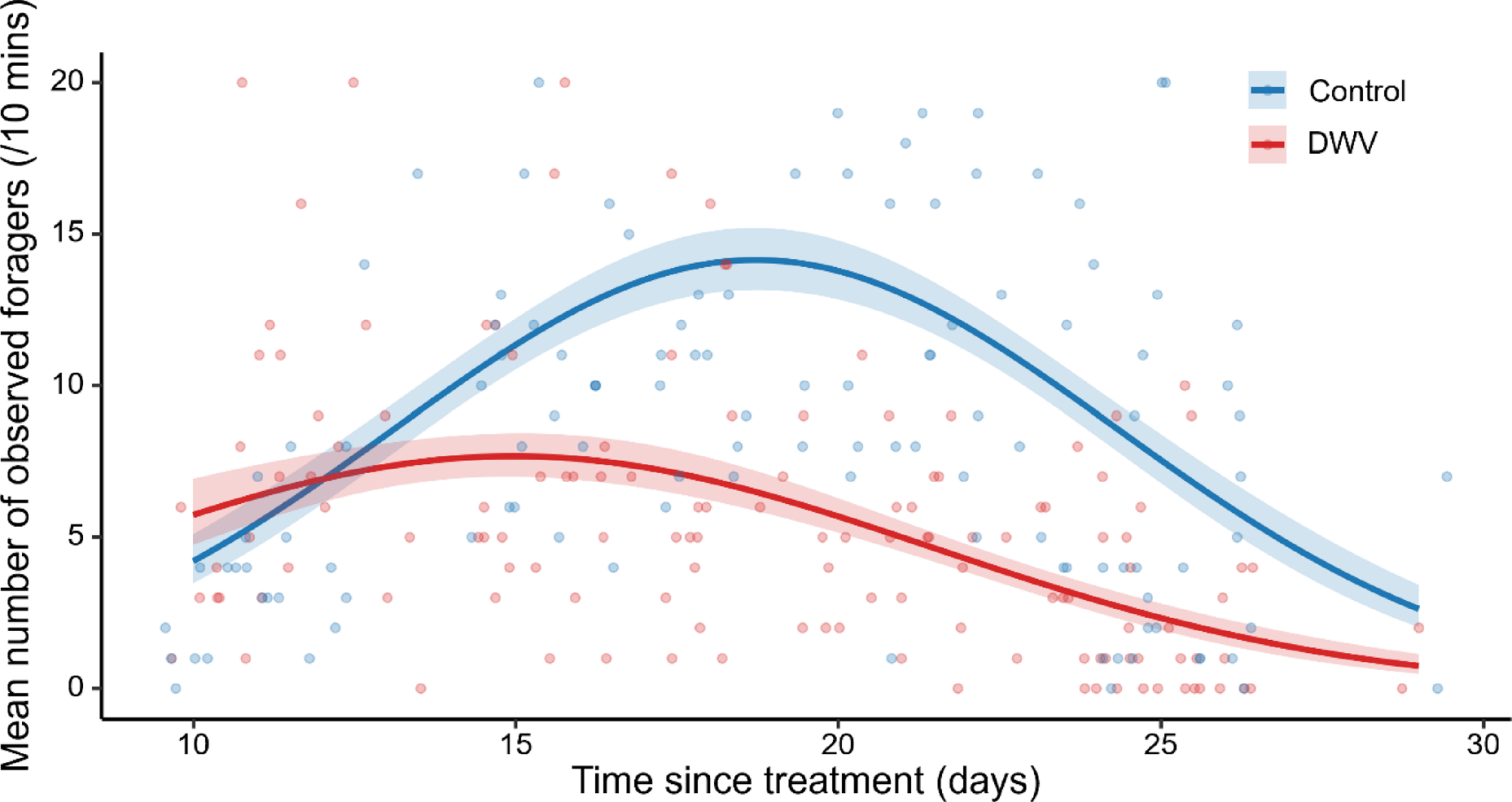
Effect of deformed wing virus (DWV) on the overall foraging activity. Mean number of observed foragers (per 10-minute interval) from control and DWV-infected colonies over a 19-day observation period. DWV-infected bees exhibited significantly lower foraging activity compared to controls (total of 2140 observed foragers from 134 daily 10-minute observation intervals across 4 colonies; Poisson GLM, *χ*^*2*^ = 199.76, *p* = 2E-45. The model included ‘colony’, ‘time of day’, ‘weather’ and interaction between ‘treatment’ and quadratic ‘time since treatment’ as fixed factors. For statistical detailed output, see Supplementary Table S1. This result indicates that DWV infection in adult workers negatively affected foraging, either through reduced foraging rates, increased mortality or both. Thick lines represent mean model predictions, with blue for control and red for DWV-infected bees, with matching shaded areas indicating their respective 95% confidence intervals. Individual coloured dots are the raw observed data points.

During the three daily 10-minute observation intervals, the foraging activity of control and DWV-infected workers was monitored over the course of the experiment (Fig. 1). Foraging activity began on day 10 after treatment and was monitored for approximately 10 days across four colonies, yielding data from 361 returning foragers. Overall, significantly fewer foragers from the DWV-infected treatment group were detected compared to control workers (Poisson GLM, *χ*^*2*^ = 199.76, *p* = 2E-45; for detailed statistical output and estimates, see Supplementary Table S1a, b), demonstrating the negative impact of DWV infections on the foraging activity of bees in the adult life stage. This reduction could be due to decreased foraging rates among affected foragers or increased mortality in the DWV-treated bees. The model also showed a significant effect of age (or day since treatment) (*χ*^*2*^ = 218.04, *p* = 5E-48; see Supplementary Table S1a) as well as the interaction between treatment and age (*χ*^*2*^ = 86.19, *p* = 2E-19; see Supplementary Table S1a). While we found a significant effect of weather (*χ*^*2*^ = 30.14, *p* = 4E-08; see Supplementary Table S1a), there was no significant interaction with treatment, indicating that the effect of weather on foraging activity that presented slightly higher levels of activity under better meteorological conditions, was similar for both treatments. Finally, the time of day, did not have a significant effect on daily foraging activity (χ² = 1.42, *p* = 0.23; see Supplementary Table S1a & S1b).

**Fig. 2:**
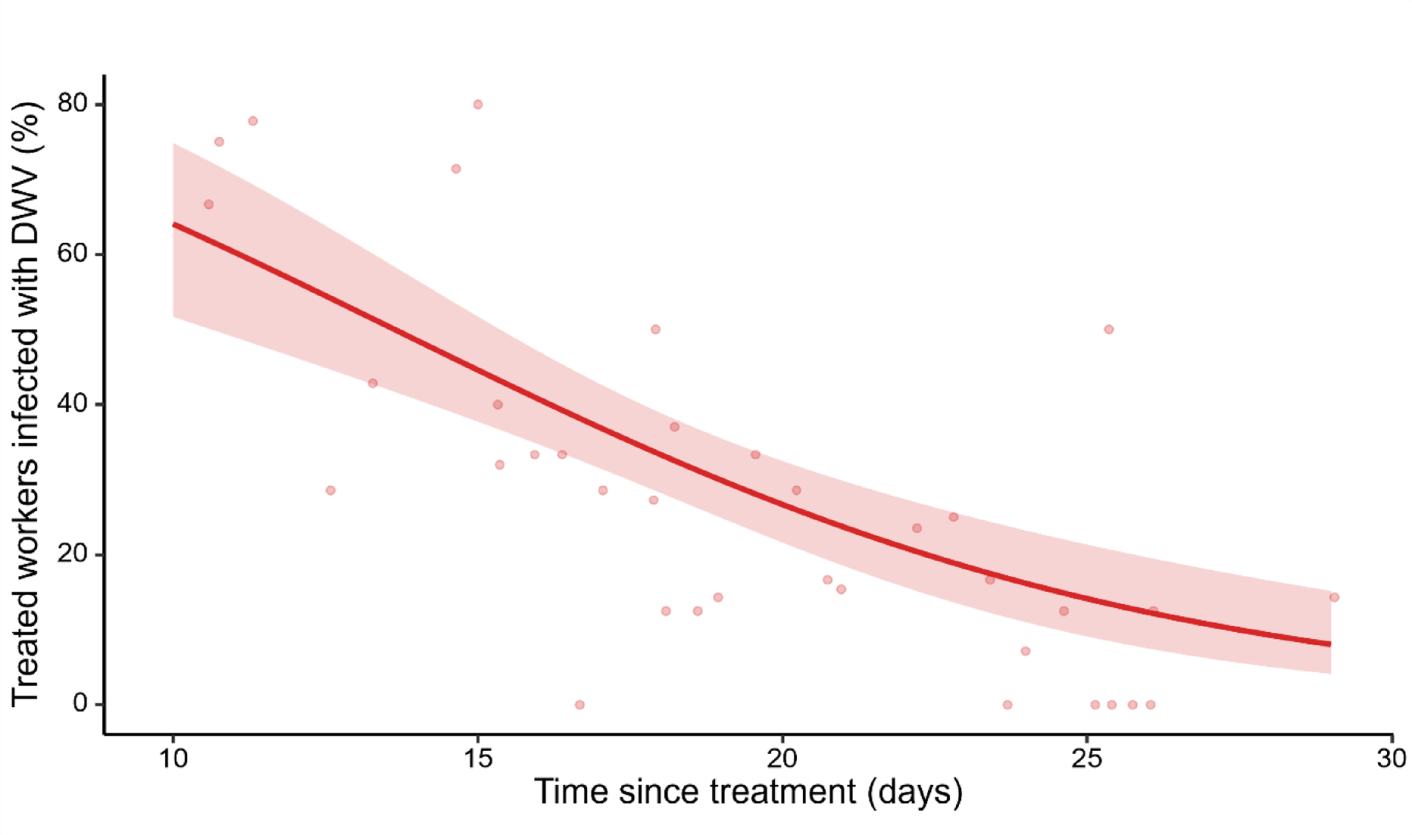
Proportion of DWV-infected adult workers over time. Initially, DWV-infected foragers constituted approximately 65%, of the observed population, which decreased significantly to less than 10% by the end of the 19-day observation period. This decline is consistent with hypotheses of DWV induced precocious foraging and subsequent virus-induced mortality in later stages of the experiment. Proportions (*n* = 40) were calculated from the sum of daily observed foragers (*n* = 361) across 4 colonies. A Binomial GLM was used, with ‘colony’ and ‘time since treatment’ as main fixed factors. The significance of the decline as a function of time since treatment was *χ*^*2*^ = 30.90, *p* = 3E-8, for detailed statistical output, refer to Supplementary Table S2. The red line shows the mean model predictions, the red shaded area represents the 95% confidence interval, and the pale red dots represent the raw data points.

We also examined the proportions of DWV-treated individuals among the total observed foragers per day throughout the experiment (Fig. 2). Although bees from both treatment groups were introduced into the colony in equal numbers, the proportion of DWV-infected foragers was notably higher early in the experiment, reaching approximately 65% of the foraging workers, before steadily decreasing over time. Around day 15, the ratio of DWV-infected to uninfected bees returned to approximately 50/50. By the end of the experiment, DWV-treated individuals comprised less than 10% of the foraging bees. This trend aligns with DWV inducing precocious foraging behaviour and with the virus reducing forager return rates and causing mortality in later stages of the experiment (Poisson GLM with ‘colony’ and ‘time since treatment’ as fixed factors, using the proportion of DWV-infected foragers as the response variable: *χ*^*2*^ = 30.90, *p* = 3E-8, see Supplementary Table S2a & S2b).

### Effect of deformed wing virus on foraging success, efficiency and specialization

The daily collection of marked foragers at the hive entrance, after a 15-minutes period during which the entrance was closed, allowed us to determine the foraging success of infected and uninfected foragers for either nectar or pollen. By measuring the sugar content of the collected nectar and weighing the collected pollen clumps, we also assess the performance of treated foragers.

**Fig. 3:**
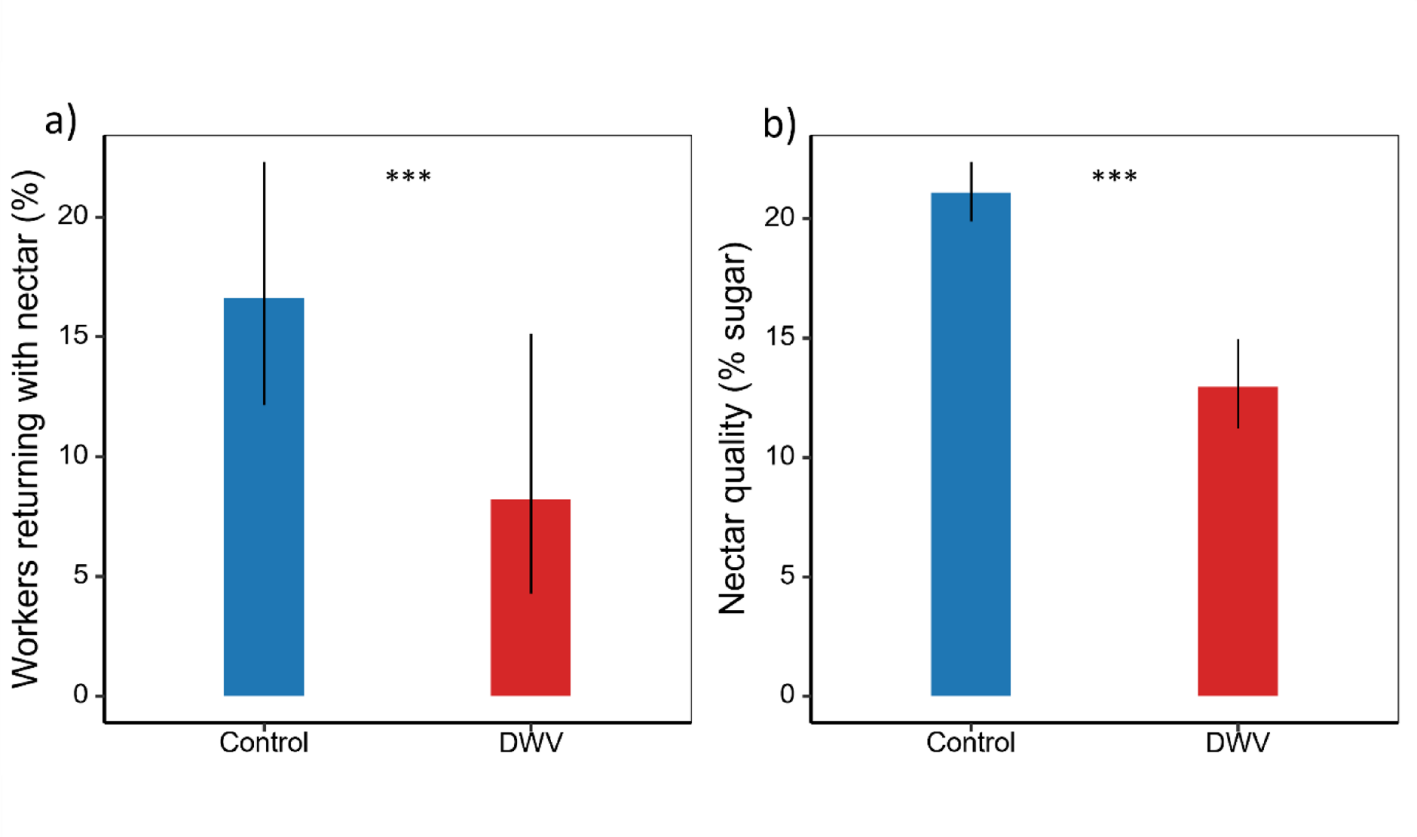
Effect of DWV on nectar foraging success and performance. **a**) Mean proportions of DWV-infected and uninfected (control) foragers returning with nectar (*n* = 57). DWV-infected foragers were significantly less likely to return with nectar than control (*χ*^*2*^ = 4.56, *p* = 0.03). Statistical analyses were based on binomial GLMs in which ‘colony’, ‘treatment’ and ‘time since treatment’ as fixed factors. For detailed statistical output, see Supplementary Table S4. **b**) Average sugar concentration (% sugar) of the nectar collected by returning foragers. Nectar collected by DWV-infected bees contained significantly less sugar concentrations than that collected by controls (χ^2^ = 38.35, *p* = 6E-10). Statistical analyses were based on linear mixed models (LMMs) of the log₁₀-transformed sugar concentration, with ‘colony’, ‘treatment’, and ‘time since treatment’ as fixed factors and ‘individual bee’ identity as a random factor. For detailed statistical output, see Supplementary Table S5. Error bars represent standard deviations.

While DWV infection had no effect on the overall foraging success of honeybees (*χ*^*2*^ = 0.003, *p* = 0.96, binomial GLM with ‘colony’, ‘treatment’ and ‘time since treatment’ as main effects, for detailed statistical output, see Supplementary Table S3a & S3b), the distribution of nectar and pollen foragers differed between treatments. DWV-infected bees were significantly less likely to return to the hive with nectar and more likely to return with pollen compared to uninfected individuals, indicating a shift in foraging specialization rather than a reduction in the total foraging success (Fig. 3a, *χ*^*2*^ = 4.56, *p* = 0.03, binomial GLM with ‘colony’, ‘treatment’ and ‘time since treatment’ as main effects, for detailed statistical output see Supplementary Table S4a & S4b). Specifically, the 16.61% probability of control bees returning with nectar, contrasts with the significantly lower 8.20% predicted for DWV-Infected bees (see supplementary Table S8).

Moreover, the nectar collected by DWV infected bees contained significantly lower concentrations of sugars compared to that collected by control foragers (Fig. 3b, χ^2^ = 38.35, *p* = 6E-10, linear mixed model analysis of log10 transformed sugar content with ‘individual bee’ as a random factor and ‘colony’, ‘treatment’ and ‘time since treatment’ as main effects; for detailed statistical output see Supplementary Table S5a & S5b). Consistent with this, the estimated mean nectar sugar content for DWV-infected bees was 12.95%, substantially lower than the 21.09% for control bees (Supplementary Table S9).

**Fig. 4:**
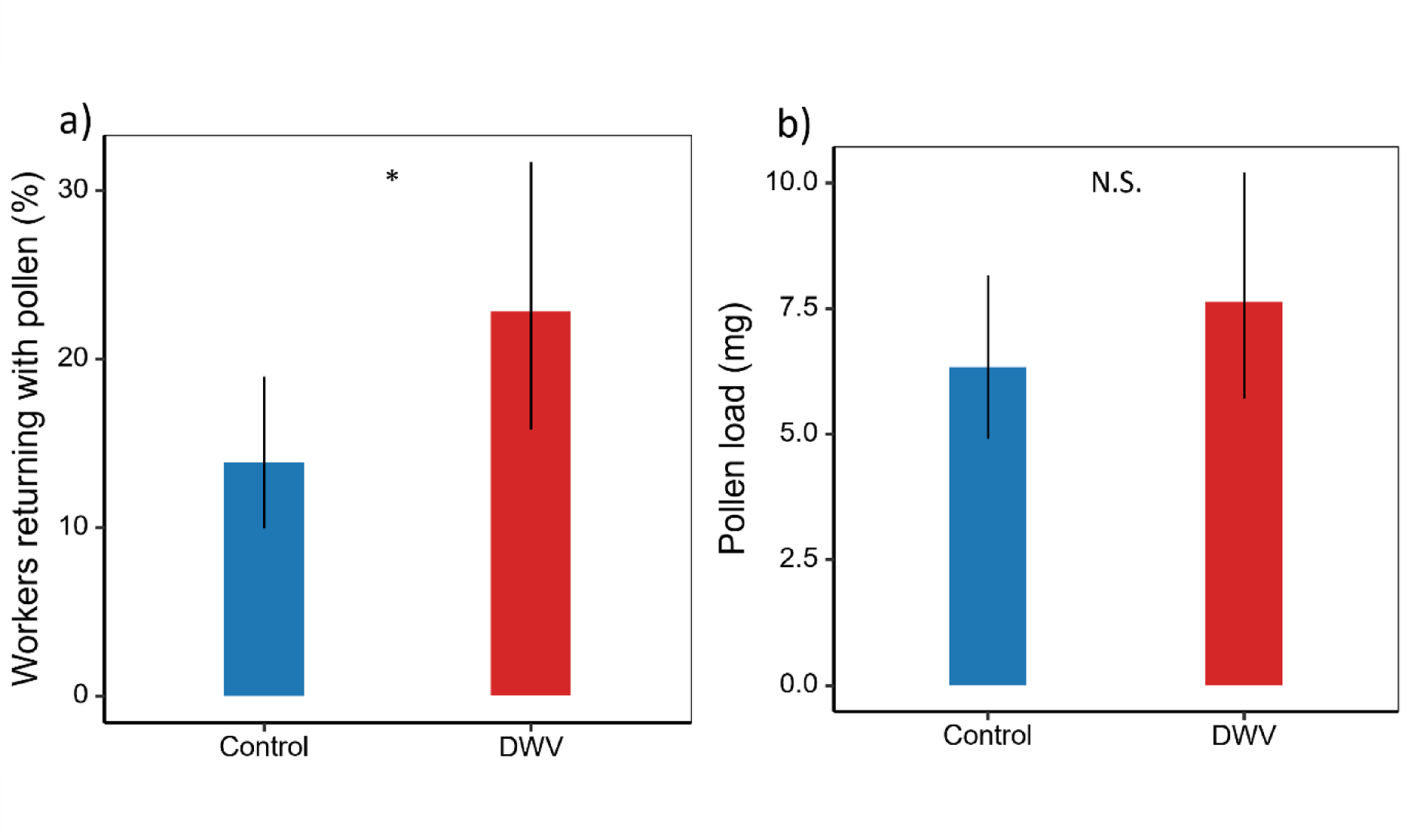
Effect of DWV on pollen foraging success and performance. **a**) Mean proportions of DWV-infected and uninfected (control) foragers returning with pollen (*n* = 62). DWV-infected foragers were significantly more likely to return with pollen than controls (*χ*^*2*^ = 3.93 *p* = 0.047). Statistical analyses were based on binomial GLMs with ‘colony’, ‘treatment’, and ‘time since treatment’ included as fixed factors. For detailed statistical output see Supplementary Table S6. **b**) Average pollen load (mg) carried by returning foragers, which did not differ significantly between treatment (χ^2^ = 0.94, *p* = 0.33). Statistical analyses were based on linear mixed models (LMMs) of log₁₀-transformed pollen load, with ‘colony’, ‘treatment’, and ‘time since treatment’ as fixed factors, and ‘individual bee’ as a random factor. For detailed statistical output, see Supplementary Table S7. Error bars represent standard deviations.

Interestingly, we found contrasting results for pollen foraging, with DWV-infected foragers being slightly more likely to return with pollen than control bees (Fig. 4a, *χ*^*2*^ = 3.93 *p* = 0.047, binomial GLM with ‘colony’, ‘treatment’ and ‘time since treatment’ as main effects; for detailed statistical output see Supplementary Table S6a & S6b). Specifically, DWV infected forager bees had an estimated probability of 22.81% of returning with pollen, compared to 13.86% for control bees (Supplementary Table S8).

However, pollen foraging efficiency was unaffected, as there was no significant difference in the weight of the pollen collected by foragers from both treatments (Fig. 4b, χ^2^ = 0.94, *p* = 0.33, linear mixed model of log10 transformed pollen loads with ‘individual bee’ coded as a random factor and ‘colony’, ‘treatment’ and ‘time since treatment’ as main effects; for detailed output see Supplementary Table S7a & S7b). The similar estimated mean pollen loads of 7.64 mg for DWV-infected bees and 6.33 mg for control bees, further corroborate that pollen foraging efficiency was not significantly impacted by the DWV (Supplementary Table S8).

The effect of DWV on the relative composition of nectar and pollen of honeybee foragers was also evident in the fact that DWV-infected foragers had approximately four times higher odds of returning with pollen rather than nectar compared to control foragers, indicating increased pollen-foraging specialization among DWV-infected foragers (Fisher exact test, *p* = 0.001, raw numbers: DWVpollen 27, DWVnectar 9, CTRLpollen 35, CTRLnectar 48).

## Discussion

Our results demonstrates that DWV infections in adult workers led to the earlier onset of foraging, (precocious foraging) and reduced overall foraging activity, which may result from direct effects on the foraging rates of infected bees, virus-induced mortality, or both. In addition, DWV-infected bees were significantly less likely to engage in nectar collection than control bees, and when they did, the nectar collected had lower sugar concentrations. This pattern, combined with the higher number of pollen foragers among DWV-infected bees, indicates a shift in foraging specialization rather than reduced foraging ability, with infected bees exhibiting significant specialization in pollen foraging. However, the mean pollen loads of pollen-collecting bees did not differ between DWV-infected and control bees. In this study, the control group was injected with a DWV-specific dsRNA to suppress potential viral replication and prevent horizontal transmission, while the DWV-infected group received a GFP-dsRNA to ensure an equivalent dsRNA treatment and maintain consistency between groups. Previous studies have shown that dsRNA exposure alone does not affect honeybee survival or foraging behaviours^49,50,52^, with GFP-dsRNA-injected bees performing similarly to non-injected controls^52^. Together, these findings indicate that dsRNA exposure itself is unlikely to have contributed to the behavioural effects observed in the infected group, which are instead associated with the DWV infection.

Daily observation of the foraging activity patterns clearly demonstrated precocious foraging behaviour, consistent with previous findings that covert DWV infections significantly impair worker foraging and reduce survival rates^49^. Furthermore, similar patterns have been documented in unhealthy and stressed workers as a response to other pathogens and parasites, such as *Nosema*^53–58^, sacbrood virus^59^ and *Varroa*^60–62^. Interestingly, recent studies have shown that the brain gene expression profiles of DWV infected bees closely mirror those of foragers, even in individuals significantly younger than typical foragers^63^.

Our findings of reduced return rates and shortened lifespan in DWV-infected foragers reinforce the growing consensus that secondary DWV infections should no longer be regarded as covert and asymptomatic^26,36,39,40,64,65^ but pose a significant threat to individual worker survival. Early onset of foraging and decreased longevity undermine colony resilience by depleting the nurse bee population^66,67^ and destabilizing colony structure, thereby impairing critical colony functions, including brood care and cell cleaning comb construction, queen attendance, thermoregulation, food processing and defense^68–71^.

Beyond its effects on foraging activity and worker survival, our results show that DWV negatively impacts the foraging efficiency of nectar collecting workers. A given volume of high-sugar nectar yields greater energetic value to the colony than the same volume of low-sugar nectar, so honeybee foragers are expected to target nectar sources with high sugar content^72^, making sucrose concentration a good proxy for foraging performance. While the sugar concentrations observed in our study are on the lower side of the spectrum^73^, such values are not uncommon during the late summer and autumn, when nectar-rich floral resources become scarce^74^. In this context, the nearly twofold difference in nectar sugar concentration collected between DWV infected and uninfected bees is striking. This finding aligns with Iqbal and Mueller^47^, who reported that DWV infections increase responsiveness to low concentration sucrose solutions. Earlier studies have also shown that foragers with lower response thresholds tend to collect nectar with lower sugar content than those with higher response thresholds^75,76^. Consistent with this, DWV-infected bees in our study were more likely to collect pollen rather than nectar, suggesting a shift in foraging specialization. Interestingly, this pollen bias mirrors patterns seen in healthy individuals with naturally lower sucrose response thresholds^76–78^, a specialization that has been linked to precocious foraging through shared neurogenetic regulation and variation in gene expression pathways underlying these traits^79–81^. Therefore, DWV may induce neural changes resembling those that naturally bias workers toward pollen foraging. Supporting this, Lemanski, et al^82^., reviewed evidence showing variation in sucrose responsiveness and foraging preference is associated with differential expression of genes involved in biogenic-amine signalling, metabolism and neural plasticity in the mushroom bodies, pathways that are also known to be modulated by stress, nutrition and pathogen exposure, which can accelerate behavioural maturation and alter sensory responsiveness. Such virus-induced disruption of neural signalling and plasticity could therefore mimic or exaggerate natural variation in foraging specialization. Iqbal and Mueller^47^ found that adult honeybees infected with DWV exhibited poor memory retention. Since spatial memory is crucial for orientation, navigation and homing^83,84^, such deficits could explain the reduced foraging success observed in infected individuals. Consistent with this framework, Pizzorno, et al^85^., showed recently showed that DWV infection leads to significant changes in gene expression in the honeybee brain, including the upregulation of immune-related genes and down regulation of genes involved in neuronal signalling, which may underlie such behavioural changes. Similar effects on foragers’ sucrose responsiveness and homing ability have also been documented in bees infected with a different virus, IAPV^48^, suggesting that viral infections more broadly can disrupt key cognitive and sensory functions in foragers.

The overall colony cost of DWV infections, through the decreased nectar yield, can be considerable. Although many beekeepers supplement colonies with sugar-water during times of dearth or after harvest, natural nectar foraging remains critical for optimal nutrition, winter preparations and brood development and is the primary carbohydrate source providing the energy required for flight and daily survival^86^. Beyond immediate energy needs, nectar is stored and transformed into honey, serving as the colony’s winter food reserve and as a nourishment for brood development. In temperate climates, small colonies may consume at least 20 kg of honey between April and July^87^. Brood rearing is particularly energy-intensive, with the development of a single worker larva requiring approximately 59.4 mg of sugar, accounting for direct larval nutrition and worker activities such as comb building, thermoregulation and nursing^88^. The energetic cost is especially high during winter brood rearing, which is particularly relevant given the timing of this study. Colonies can lose around 0.84 kg per week during this period, nearly double the 0.42 kg lost per week when no brood is being reared^87^. DWV infection can exacerbate these challenges as even covert infection have been shown to reduce foraging efficiency and worker longevity, diminishing the colony’s ability to collect and store nectar in preparation for winter^49^. Reduced nectar collection before winter may limit the buildup of honey reserves and brood-rearing capacity, particularly in regions or management contexts where colony density exceeds the landscape’s floral carrying capacity. While supplemental feeding by beekeepers often buffers against food shortages, DWV-induced reduction in nectar foraging efficiency could exacerbate existing nutritional stress and contribute to winter losses under suboptimal foraging conditions.

Interestingly, we found an opposite effect of DWV on foraging specialization, with infected workers being more likely to return to hive with pollen than uninfected foragers and no significant difference in the weight of the pollen collected by foragers from both treatments. The pollen loads observed were typical for honeybees in temperate regions^89,90^, and the lack of variation between infected and uninfected bees was unexpected, given the high potential for variability in pollen foraging^89^. A possible explanation is the abundance of pollen sources near hives, which would require minimal foraging effort to obtain a consistent reward. In contrast, nectar yielding flowers were much more limited in the vicinity, as is often the case in late summer or autumn. The scarcity likely compelled nectar foragers to travel greater distances to find suitable resources^72,74^. This combination of local resource distribution altered sucrose responsiveness, and impaired homing ability due to memory deficits in infected bees may explain why DWV-infected workers tend to specialize in pollen foraging. While they appear equally efficient as uninfected bees at collecting pollen, their ability to collect high-quality nectar is significantly impaired, thereby compromising the colony’s overall nectar collection.

Our study shows that DWV infections have more harmful effects on honeybee foraging behaviour, health and colony viability than previously recognized. While overt DWV infections are known to cause visible symptoms and high mortality, our findings underscore the overlooked, yet significant, costs of covert infections during the adult life stage. These results align with growing evidence linking DWV to colony declines across Europe^17–20,22^, in the US ^23,27^ and Canada^25^, and reinforce the central role of DWV in honeybee health and colony losses. Although DWV’s effects are largely restricted to honeybees, these losses represent an important component of the broader ongoing pollinator crisis^4^. Critically, our data shows that DWV selectively impacts nectar foraging, without affecting pollen collection, potentially disrupting the colony’s ability to build sufficient honey stores and rear brood during energetically demanding period such as winter. These findings suggest a plausible mechanistic link between sublethal viral infections and seasonal colony losses, particularly under conditions of limited floral availability or high colony density. While DWV alone may not fully explain colony declines, it potentially interacts with other stressors. Given the central role of foragers in colony sustainability, even subtle reduction in foraging efficiency could have compounding, long-term impacts on population resilience. This study offers a foundation for future research into how viral pathogens interact with factors such as poor nutrition, habitat loss and pesticide exposure. Further research into how DWV affects forager orientation, endurance in long distance flights, sensory processing and decision-making under varied environmental conditions will be essential to fully understand its effects on honeybee health and behaviour. As DWV continues to spread globally, understanding its sublethal consequences is not only relevant for colony management but also for broader ecosystem stability and food security.

## Materials and methods

### Observation set up and experimental design

**Fig. 5:**
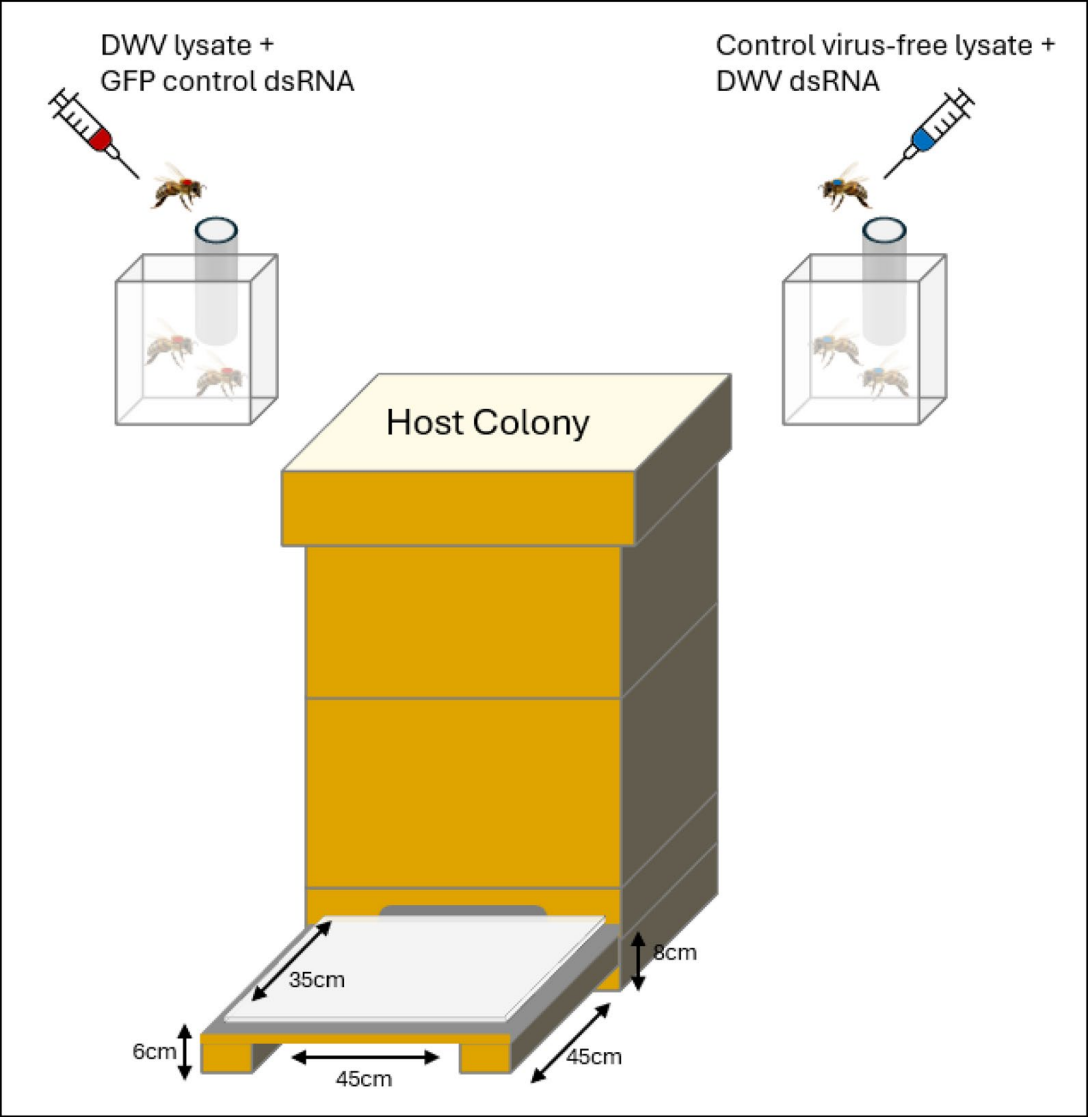
Experimental setup used to monitor foraging behaviour of deforming wing virus (DWV) infected and control bees. Bees were subjected to one of the two treatments: (1) DWV lysate + GFP control dsRNA (top left) or (2) Control virus-free lysate + DWV dsRNA (top right). Marked bees, identifiable by two different colour codes corresponding to the treatment, were introduced into the host colony via separate cages (top section of the diagram). A total of 500 bees per treatment were introduced into each of four host colonies (two colonies per replicate, replicated twice). A custom-made bottom board with a Plexiglas cover, whose key dimensions are indicated, was utilized to facilitate direct observation of marked bees entering and exiting the hive. Observations of marked foragers were made for 10 min, three times a day, over a period of 26 days.

During the summer and autumn of 2013, experiments were carried out twice (two independent replicates within the same year), each time using two different host colonies (4 in total), set up at the apiary of the laboratory of Socioecology and Social Evolution (LSSE) in Leuven, Belgium (50° 52′ 46′′ N; 4° 42′ 3′′ E). Colonies were housed in traditional Belgian Simplex beehives, each consisting of two brood boxes with eleven frames. The hives were equipped with an extended Plexiglas-covered bottom board to allow for the observation of the foragers entering and leaving (Fig. 5). In addition to the experimental hives, four to six non-experimental colonies were present at the site.

In each of the forementioned host colonies, we introduced 500 DWV-negative control bees and 500 DWV infection-positive honeybees. This was done by allowing bees to emerge from a single virus-free donor colony, injecting newly eclosed workers with appropriate treatment solutions (see below for the detailed methods: *Controlled infection*), and introducing these bees into each host colony. The virus-free status of this donor colony was confirmed at the start of the experiment by screening 20 randomly selected workers using multiplex ligation-dependent probe amplification (MLPA)^91^, confirming the absence of DWV and other common honeybee viruses. Because both treatment groups were introduced into the same host colonies, DWV- specific dsRNA was added to the control inoculum to suppress any potential viral replication and prevent horizontal transmission from infected to control bees^49,50^ (see below for the detailed methods: *Controlled infection*).

To obtain the newly emerged bees for inoculation, brood frames from the donor colony were placed in an incubator (MIR-253, Sanyo, Belgium) at 34 °C and 60% humidity. Newly eclosed workers were collected daily. A total of 500 newly eclosed workers per treatment condition and host colony were injected with 3 µl of the corresponding treatment solution (see below), using a 5 µl, 26s-gauge Hamilton syringe (Microlitertm Hamilton), carefully inserted into the apical part of the thorax. Microinjections using fine Hamilton syringes is widely used and well-tolerated procedure in honeybee research and does not cause lasting effects on survival^49,92–94^, and both treatment groups were injected identically, ensuring that any transient handling stress was equally distributed. Immediately after injection, each bee was individually marked with a numbered Opalith tag using shellac adhesive (Bijenhof, Belgium) with different colours corresponding to the different treatment groups and colonies. Groups of 50 individuals that were subjected to one of the two treatments were housed in separate cages (15 × 10 × 7 cm) under controlled conditions (34 °C and 60% humidity), with access to a 10 × 8 cm piece of honey-filled comb and drinking water. This allowed the bees to settle down prior to their introduction into the host colonies. For introduction, cages were placed inside the hives and opened after a 30-minute period to improve acceptance rates.

The experiments ran for 26 days following the introduction of the treated bees. Each experimental colony was observed for 10 min, three times daily (between 9:00 and 10:00 am; 12:00 am and 1:00 pm; 4:00 and 5:00 pm) excluding periods with a moderate to heavy rainfall, during which bees reduce or even cease foraging activity. All marked individuals entering and leaving the hive during the observation window were tallied and grouped in a single forager activity variable. This resulted in a first dataset consisting of the activity of the observed foragers, their corresponding age, treatment, and colony, as well as information on the weather and the time of day at sampling. Foraging success was defined as the proportion of returning bees carrying nectar or pollen out of all observed foragers. Foraging efficiency (here used synonymously with “foraging performance”) was defined as a measure of resource quality and quantity, based on nectar sugar concentration and pollen load mass. Foraging specialization was calculated as the proportion of successful foragers returning with pollen relative to all successful (food-carrying) foragers, thereby distinguishing foraging choice from overall foraging success. Weather information was categorized post hoc into three levels: above average, average, and below average.

Every non-rainy day at 3:00 pm, hive entrances were closed for 15 min, after which all marked individuals present at the hive entrance were collected in individual 50 ml Falcon tubes for subsequent pollen and nectar collection. To facilitate handling, honeybees were briefly anesthetized by placing the Falcon tubes at 4 °C. Individual identification (colour and number of Opalith disc) was recorded to link each bee to its respective nectar and pollen content data.

### Controlled infection

To obtain two groups of adults, age-matched bees that were either infected with DWV or not infected for our experimental observations, we first prepared two treatment solutions. These solutions were designed to either increase DWV virus titers or to serve as a control solution.

This required the preparation of two different lysates: one from honeybees infected with DWV but free of other common honeybee viruses, and another from honeybees confirmed to be free of DWV and any other virus. To do this, we screened 10 individual bees from each of 10 *A. mellifera carnica* colonies in our apiary, as well as from screening individuals displaying overt symptoms of DWV-infection (i.e., crippled wings). Based on MLPA analysis^91^ to determine the DWV infection status and the use of specific PCR primers, we selected suitable samples and created two pools of 10 bees each: one for the DWV-infected group and one for the virus-free control.

From each of these pools, we extracted the lysates by homogenizing tissue corresponding to five bees (around 500 mg), which had been stored at −80 °C. After immersion in liquid nitrogen, the tissue was homogenized in 5 ml of phosphate-buffered saline (PBS, pH 7.4). The samples were then centrifuged at 3000 r.p.m. for 30 min at 4 °C, and the resulting supernatant was stored in aliquots at −80 °C for future use^47^. The artificial inoculation with a DWV lysate used in this study has previously been shown to be effective in short-term, individual-based setups^47^. In the present study, however, the bees remained in close contact with each other in the observation hive throughout their adult lives, allowing the virus to potentially be transmitted horizontally during the experimental period. To mitigate the effects of horizontal transmission from DWV-infected bees to the uninfected group^44^, we opted to include a double-stranded RNA (dsRNA) treatment in our DWV negative lysate (control lysate)^50,95^. DWV-specific dsRNA was added to the control lysate to maintain these bees as DWV-free and prevent cross-infection of DWV among honeybees within the host colony. Meanwhile, control Green Fluorescent Protein (GFP)-dsRNA was added to the DWV lysate to control for any potential physiological effects of foreign dsRNA injection on honeybee health. DWV specific dsRNA and a GFP-dsRNA were provided by Beeologics (Israël) and were dissolved in nuclease free water at 3 mg/ml. Prior to injection, both DWV and control lysates were diluted 1:1000 (cf. Iqbal and Mueller^47^ in insect saline buffer (ISB, containing 150 mM NaCl, 10 mM KCl, 4 mM CaCl2, 2 mM MgCl2, and 10 mM HEPES; pH 7.0). The final treatment solutions were prepared by adding dsRNA at a dosage of 5 µg per bee. Infection status after injection was inferred from the use of validated DWV-positive and virus-free lysates and post-treatment viral titers were not quantified in the experimental bees and the individual infection. Throughout the experiment, all bees were visually monitored for overt symptoms of DWV, such as shrivelled or deformed wings. No individuals displaying such visible deformities were observed. To evaluate the effects of these infections on the overall foraging behaviour and resource quality, we analysed the nectar and pollen collected by treated individuals.

### Nectar and pollen collection

The contents of the bee’s crop were obtained by gently applying pressure to the abdomen with a piece of polystyrene foam between thumb and index finger, mainly to avoid being stung. The expelled liquid was collected on the prism face of a refractometer (HR25/800, 21 °C; Krüss, Belgium) to measure the sugar concentration of the nectar. Using forceps, pollen was gently collected from the hind legs and placed into pre-weighed Eppendorf tubes. The tubes were then weighed again using a precision laboratory scale (Secura; Sartorius, Germany), and the actual pollen load was calculated by subtracting the initial tube weight from the final weight. After sampling, honeybees were allowed to recover in the collection tubes before being released back at the respective hive entrance.

### Ethical note

*Apis mellifera* is a common, non-endangered species in Belgium. Nevertheless, bees were delicately collected and promptly transported to the laboratory, where bees were carefully managed under controlled CO₂ exposure to minimize stress during handling. All conditions for animal welfare were ensured, and at the end of the experiment, bees were humanely euthanized by freezing at − 20 °C. Only the necessary number of colonies and individuals were used to achieve robust statistical analyses, in line with the principles of reduction, refinement, and replacement. Field sampling and experiments with live bees were conducted in accordance with Belgian and European regulations for animal experimentation (Belgian Royal Decree of 6 April 2010 and European Directive 2010/63/EU on the protection of animals used for scientific purposes of 20 October 2010).

### Data analysis

To assess trends in the overall foraging behaviour of DWV infected and control bees, data from daily observations were analysed. Overall foraging activity was modelled using Poisson distributed generalized linear models (GLM’s), with ‘treatment’, ‘day since treatment’ (using poly(DAY_SINCE_TREATMENT, 2) for age effect), ‘time of day’ and ‘weather’ and ‘colony’ as fixed factors. The final model was selected based on the Akaike Information Criterion (AIC) values to minimize information loss. This model included the interaction between ‘treatment’ and ‘day since treatment ‘, but no other interactions between the variables. To thoroughly investigate the effects of DWV infection on the foraging behaviour of honeybee workers, we examined both their overall foraging success (the proportion of returning bees carrying any food) and their foraging specialization (the proportion of successful foragers returning with pollen versus nectar). For each instance of foraging success as the dependent variable we used binomial GLMs with ‘colony’, ‘treatment’ and ‘day since treatment’ as fixed factors. To test whether DWV infection affected the yield of foraged nectar and pollen, we modelled the sugar content in collected nectar and the weight of collected pollen. Both analyses were conducted using linear mixed models (LMMs) with ‘individual bee ' identity as random factors and ‘treatment’, ‘day since treatment’ and ‘colony’ as fixed factors. Because only four colonies were used in total, colony was included as a fixed factor in all analysis to explicitly control for potential colony-level variation. With so few groups, estimating colony as a random effect would yield unreliable variance estimates and frequently lead to convergence issues or model overparameterization^96,97^. In the nectar quality and pollen load analyses, ‘individual bee identity’ was included as a random effect in the LMMs to account for withing-colony variability. All statistical analyses were performed using R v. 4.3.3 (R Core team, 2025). Model fitting was conducted using the *lme4* (v. 1.1–9.1) package for mixed-effects models and *stats* (v. 4.3.3) package to generate effect plots. For analyses involving generalized linear models, the *stats* package was used for model fitting. Visualization and effect plots were generated using the *ggplot2* (v. 3.5.1) and *effects (*v. 2.3-0.3) packages, respectively.

## Supplementary Information

Below is the link to the electronic supplementary material.

Supplementary Material 1

## Data Availability

The datasets generated and/or analysed during the current study are available in the Mendeley Data repository, DOI: 10.17632/ngw4bhd7mp.1.
